# Phytochemical Characterisation and Bioactivity of *Picnomon acarna* Extracts: LC–MS/MS Profiling, Antioxidant Capacity and Enzyme Inhibition

**DOI:** 10.3390/molecules31081240

**Published:** 2026-04-09

**Authors:** Zeyneb Karakus

**Affiliations:** Chemical Technology Program, Çay Vocational School, Afyon Kocatepe University, Afyonkarahisar 03700, Türkiye; zkarakus@aku.edu.tr

**Keywords:** *Picnomon acarna*, phenolic profiling, LC–ESI–MS/MS, antioxidant activity, enzyme inhibition, acetylcholinesterase, molecular docking

## Abstract

*Picnomon acarna* (L.) Cass. is a Mediterranean medicinal plant with limited phytochemical and bioactivity characterisation. In this study, methanolic extracts obtained by maceration (MAC), Soxhlet (SOE), and ultrasound-assisted extraction (UAE) were comparatively investigated to determine their phytochemical composition and biological potential. Liquid chromatography–electrospray ionisation–tandem mass spectrometry (LC–ESI–MS/MS) analysis identified and quantified 24 phenolic compounds, with hesperidin, chlorogenic acid, and hyperoside as the dominant constituents. The maceration extract exhibited the highest total phenolic content (29.06 mg GAE/g extract) and showed superior antioxidant performance across six complementary assays [2,2-diphenyl-1-picrylhydrazyl (DPPH), 2,2′-azinobis-(3-ethylbenzothiazoline-6-sulfonic acid) (ABTS), cupric reducing antioxidant capacity (CUPRAC), ferric reducing antioxidant power (FRAP), phosphomolybdenum, and ferrous-ion chelation), reflected by the highest relative antioxidant capacity index (RACI = 0.93). Enzyme inhibition assays revealed extraction-dependent activity patterns: Soxhlet and ultrasound extracts demonstrated stronger acetylcholinesterase inhibition (IC_50_ ≈ 1.23 mg/mL), while Soxhlet extract showed the most potent tyrosinase (AChE) inhibition (IC_50_ = 1.48 mg/mL). α-Amylase inhibition was comparable among extracts (IC_50_ = 1.90–2.03 mg/mL). Pearson correlation analysis indicated strong relationships between major phenolics and antioxidant activity. Molecular docking further supported these findings, showing favourable binding affinities of hesperidin, hyperoside, and chlorogenic acid toward α-amylase and acetylcholinesterase, while only chlorogenic acid and hyperoside demonstrated favourable interactions with tyrosinase-related protein-1 (TYRP1), whereas hesperidin did not exhibit a meaningful binding affinity. Overall, the results demonstrate that the extraction strategy significantly influences the phenolic composition and multi-target bioactivity of *P. acarna*, highlighting its potential as a source of natural antioxidant and enzyme-modulating compounds.

## 1. Introduction

Medicinal plants remain an indispensable reservoir for modern pharmacotherapy. The World Health Organization estimates that traditional or herbal medicine still supplies primary health care for about 80% of the global population [1]. Reflecting this societal reliance, a survey of small-molecule approvals between 1981 and 2019 showed that >50% were either natural products or their direct derivatives, underscoring the continuing translational power of botanical chemistry [2].

Oxidative stress is a recognised driver of chronic, degenerative, and metabolic diseases, and phenolic-rich botanicals are among the most studied natural antioxidants. Meta-analysis of recent data confirms that radical-scavenging and metal-reducing capacities scale with total phenolic and flavonoid loads across diverse taxa [3]. Within the Cardueae tribe, cypsela extracts of *Picnomon acarna* displayed some of the highest phenolic titres measured to date, translating into pronounced DPPH, FRAP, and CUPRAC responses [4]—evidence that this species may be a valuable redox modulator. In a broader biomedical context, oxidative stress is strongly implicated in the pathogenesis of several chronic disorders, including neurodegenerative diseases such as Alzheimer’s disease, metabolic syndromes such as diabetes mellitus, and skin disorders associated with abnormal melanogenesis. Accordingly, antioxidant and enzyme inhibitory activities—particularly against acetylcholinesterase, α-amylase, and tyrosinase—are widely investigated as complementary strategies for managing these conditions.

Beyond redox regulation, plant metabolites frequently target key enzymes involved in human pathologies. Flavonoids, stilbenes, and related phenolics inhibit tyrosinase, offering leads against hyperpigmentation and food browning [5]. Natural α-amylase blockers from edible and medicinal plants can delay post-prandial hyperglycaemia, providing safer adjuncts to current anti-diabetic drugs [6]. Meanwhile, multiple flavonoid scaffolds also antagonise acetylcholinesterase, positioning botanicals as promising multi-target candidates for neurodegenerative disorders [7]. However, the efficiency of these biological activities is closely linked to the extraction methodology employed. Extraction techniques such as maceration, Soxhlet extraction, and ultrasound-assisted extraction differ significantly in terms of temperature, solvent penetration, and mass transfer dynamics, which in turn influence both the yield and chemical composition of bioactive compounds. Previous studies have demonstrated that phenolic compounds, particularly flavonoids, are sensitive to extraction conditions, with elevated temperatures leading to significant degradation and reduction in total flavonoid content [8]. Moreover, although increased energy input may enhance mass transfer, it can simultaneously accelerate the degradation of bioactive compounds [9], and ultrasound treatment itself has been shown to induce degradation of certain phenolic acids [10]. Therefore, comparative evaluation of extraction strategies is essential for optimising the recovery of high-value phytochemicals and maximising bioactivity.

*Picnomon* Cass. is a monotypic genus represented by *P. acarna* (L.) Cass., a spiny annual native to the eastern Mediterranean and south-western Asia. Early phytochemical work isolated gossypetin-trimethyl ether and methylated luteolin congeners from its aerial parts, revealing a flavone-rich chemotype [11]. More recently, cypsela extracts ranked among the phenolic-richest members of Cardueae and expressed noteworthy antioxidant capacity [4]. However, no study has yet compared different extraction technologies, generated a comprehensive liquid chromatography–electrospray ionisation–tandem mass spectrometry (LC–ESI–MS/MS) fingerprint, or linked the phytochemical matrix of *P. acarna* to a broad antioxidant and enzyme-inhibitory panel.

Despite the ethnobotanical relevance of *P. acarna*, comprehensive studies linking extraction strategy, phytochemical composition, and multi-target bioactivity remain limited. Therefore, the present study aimed not only to comparatively evaluate methanolic extracts of *P. acarna* obtained using maceration, Soxhlet, and ultrasound-assisted extraction, but also to elucidate how different extraction strategies influence the recovery of bioactive phenolic compounds and their associated biological activities.

## 2. Results

### 2.1. Chemical Profile

**Figure 1:**
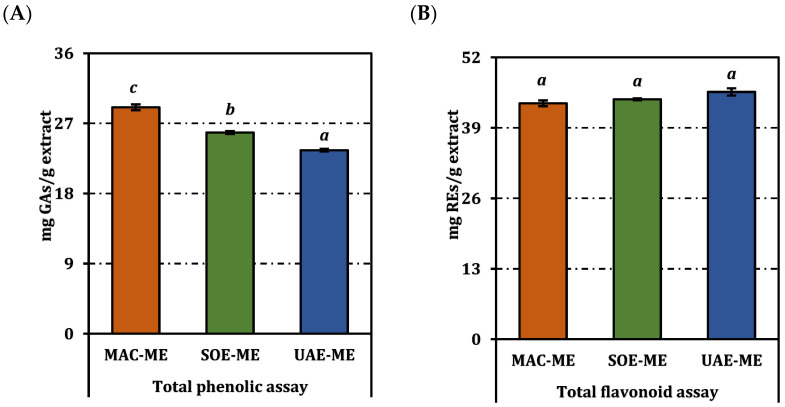
Total phenolic (**A**) and flavonoid (**B**) contents of the methanol extracts from *P. acarna*. REs and GAEs: Rutin and gallic acid equivalents, respectively. Values indicated by the same superscripts (a–c) are not significantly different according to Tukey’s post hoc test at the 5% significance level.

The total phenolic content of the three methanolic extracts differed markedly (Figure 1). Maceration delivered the highest concentration, 29.06 mg gallic-acid equivalents (GAE)/g extract, followed by Soxhlet at 25.83 mg GAE/g, and ultrasound-assisted extraction at 23.56 mg GAE/g; the distinct superscript letters denote statistically significant pairwise differences (*p* < 0.05). In contrast, total flavonoid levels were statistically indistinguishable: UAE-ME, SOE-ME, and MAC-ME yielded 45.63, 44.28, and 43.53 mg rutin equivalents (RE)/g extract, respectively, and all shared the same superscript letter. Thus, despite the generally reported efficiency of ultrasound-assisted extraction, the flavonoid yield did not differ significantly among methods, suggesting that ultrasound did not confer a measurable advantage under the applied conditions. This may be attributed to the extraction of a flavonoid pool dominated by structurally stable compounds that are readily extractable even under mild conditions.

The LC–ESI–MS/MS analysis quantified 24 phenolics in the three methanolic extracts (Table 1). Hesperidin dominated the profile, attaining 5896 µg/g in MAC-ME and dropping by roughly 20% in the ultrasound extract (4722 µg/g) and by 26% in the Soxhlet fraction (4372 µg/g). Chlorogenic acid followed the same descending order—5135 > 4139 > 3800 µg/g—highlighting the superior capacity of room-temperature maceration to conserve medium-polarity caffeoylquinates.

Among flavonol glycosides, hyperoside reached 3025 µg/g in MAC-ME but was 25% lower after Soxhlet or ultrasound extraction (≈2.31 mg/g), while the macerate also retained the greatest amounts of luteolin-7-glucoside (60.2 µg/g) and apigenin-7-glucoside (59.1 µg/g). The sharp decline of these conjugates in SOE-ME (32.8 and 42.1 µg/g, respectively) indicates partial hydrolysis under extended reflux.

Aglycone behaviour contrasted with that of glycosides. Quercetin almost doubled under Soxhlet conditions (10.56 µg/g) compared with MAC-ME (5.93 µg/g) and UAE-ME (6.75 µg/g), suggesting that heat-assisted deglycosylation liberated the free flavonol. A similar, though less pronounced, enrichment was observed for 2-hydroxycinnamic acid, which peaked at 3.19 µg/g in SOE-ME.

Low-abundance benzoic derivatives (e.g., 2,5-dihydroxy- and syringic acids) varied by <15% and shared identical superscripts, confirming that these highly polar molecules were largely extraction-indifferent. Several putative standards—pyrocatechol, (+)-catechin, verbascoside, and rosmarinic acid—remained below the detection limit (nd) in all samples.

Collectively, the quantitative fingerprint reveals clear differences in phenolic composition among extraction methods.

### 2.2. Antioxidant Activity

**Figure 2:**
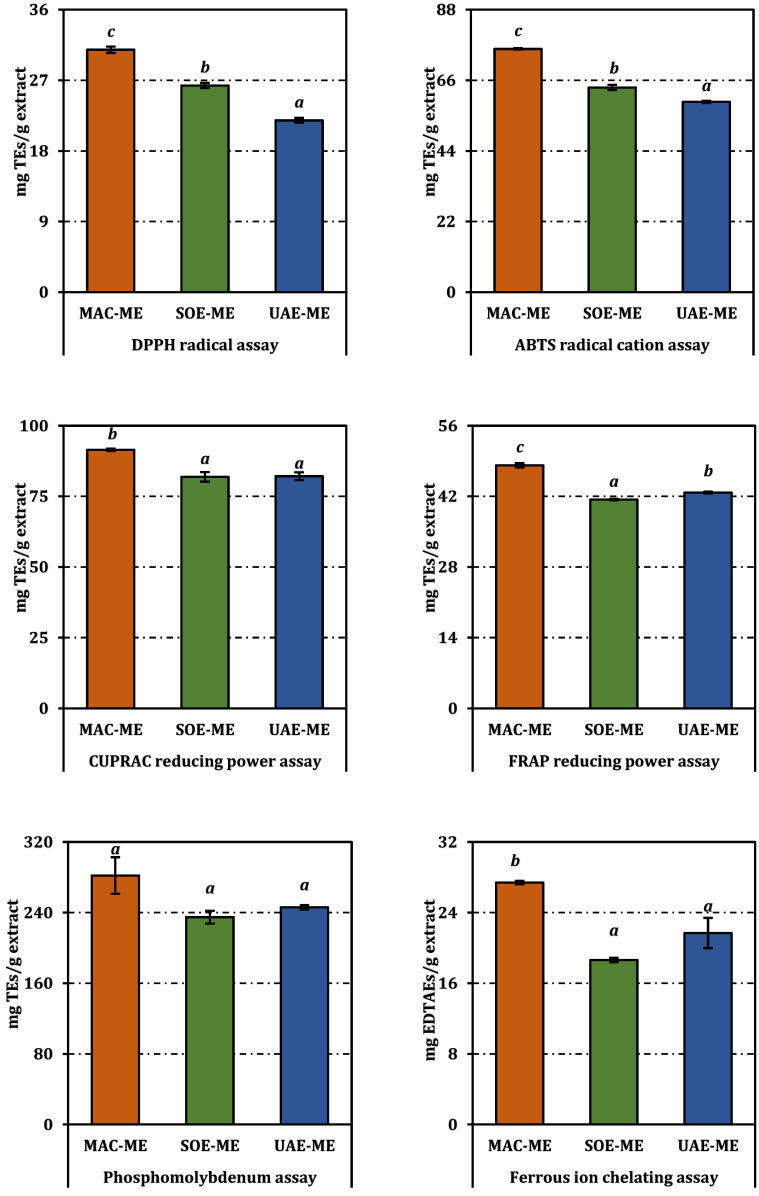
Antioxidant activity of the methanol extracts from *P. acarna*. Values indicated by the same superscripts (a–c) are not significantly different according to Tukey’s post hoc test at the 5% significance level.

All three methanolic extracts displayed measurable antioxidant potential, but their efficacies varied significantly according to both assay system and extraction protocol (Figure 2 and Table 2).

In the radical-quenching assays, the macerated sample (MAC-ME) outperformed its counterparts. It inhibited DPPH with an IC_50_ of 1.85 mg/mL, a value that was markedly lower than those for the Soxhlet (SOE-ME, 2.16 mg/mL) and ultrasound-assisted extracts (UAE-ME, 2.60 mg/mL), as indicated by non-overlapping superscript letters. A similar hierarchy emerged in the ABTS assay, where MAC-ME reached 50% scavenging at 1.45 mg/mL, compared with 1.73 mg/mL for SOE-ME and 1.86 mg/mL for UAE-ME. Although trolox remained an order of magnitude more potent (0.11 mg/mL), the values for the crude extracts are noteworthy, given their unrefined nature.

Reducing-power tests accentuated these differences. In the FRAP assay, MAC-ME registered an EC_50_ of 0.91 mg/mL, whereas UAE-ME and SOE-ME required 1.03 mg/mL and 1.06 mg/mL, respectively, to achieve the same effect. The CUPRAC method yielded a narrower spread—1.20 mg/mL for MAC-ME versus 1.34 mg/mL for SOE-ME and UAE-ME—but the superscript lettering confirmed that the differences remained statistically significant. In the phosphomolybdenum assay, which integrates electron- and hydrogen-transfer mechanisms, MAC-ME again led (EC_50_ = 1.74 mg/mL), followed by UAE-ME (1.99 mg/mL), and SOE-ME (2.09 mg/mL).

Ferrous-ion chelation mirrored the radical-scavenging data: MAC-ME exhibited the lowest IC_50_ (1.20 mg/mL), indicating superior metal-binding efficiency relative to UAE-ME (1.53 mg/mL) and SOE-ME (1.77 mg/mL). Nevertheless, all extracts were substantially less potent than the reference chelator EDTA (0.036 mg/mL).

**Figure 3:**
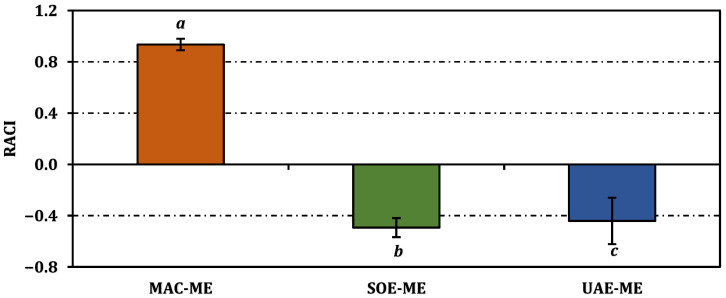
Relative antioxidant capacity index of the methanol extracts from *P. acarna.* Values indicated by the same superscripts (a–c) are not significantly different according to Tukey’s post hoc test at the 5% significance level.

The relative antioxidant capacity index (RACI) corroborated the assay-by-assay outcomes, yielding a markedly positive score for MAC-ME (0.93) and negative values for both SOE-ME (−0.49) and UAE-ME (−0.44); the distinct superscript letters confirm that maceration furnished a significantly superior global antioxidant profile (*p* < 0.05) (Figure 3).

Overall, the antioxidant assays indicate that MAC-ME consistently exhibited lower IC_50_/EC_50_ values compared to SOE-ME and UAE-ME across most test systems.

### 2.3. Enzyme Inhibitory Activity

**Figure 4:**
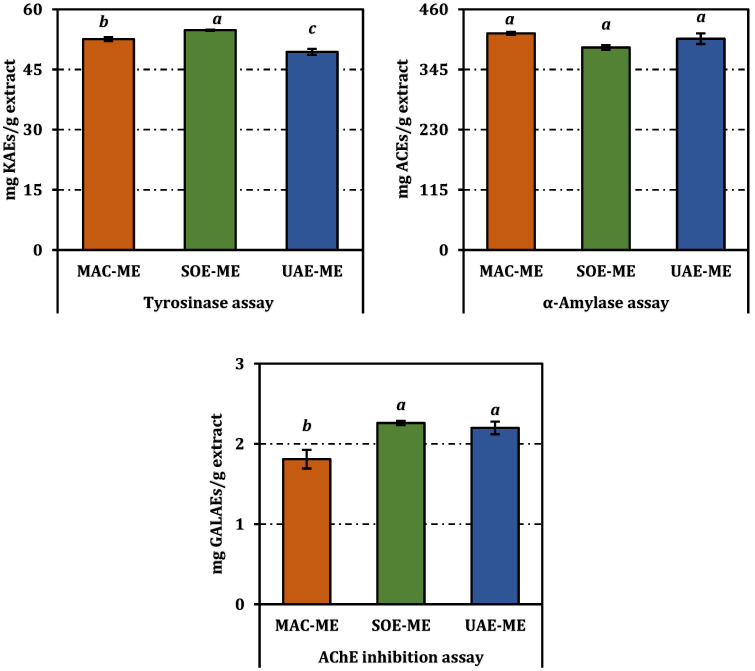
Enzyme inhibition activities of the methanol extracts from *P. acarna* expressed as acarbose (ACEs), galanthamine (GALAEs), and kojic acid equivalents (KAEs). Values indicated by the same superscripts (a–c) are not significantly different according to Tukey’s post hoc test at the 5% significance level. Enzyme inhibition activity of the methanol extracts from *P. acarna*. Values indicated by the same superscripts (a–c) are not significantly different according to Tukey’s post hoc test at the 5% significance level.

The three methanolic extracts showed discernible yet enzyme-specific inhibition patterns (Table 3 and Figure 4).

For acetylcholinesterase (AChE), SOE-ME, and UAE-ME extracts were statistically indistinguishable, presenting the lowest IC_50_ values at 1.21 mg/mL and 1.25 mg/mL, respectively. The MAC-ME was less active, requiring 1.52 mg/mL to reach 50% inhibition. Although all extracts were far weaker than the reference inhibitor galanthamine (0.0029 mg/mL).

Tyrosinase inhibition displayed an inverted hierarchy. SOE-ME again gave the most potent response (IC_50_ = 1.48 mg/mL), outperforming MAC-ME (1.54 mg/mL) and especially UAE-ME (1.64 mg/mL). While all values remain an order of magnitude higher than that of kojic acid (0.083 mg/mL), the Soxhlet extract’s superiority suggests that the thermal liberation of aglycone flavonols (e.g., quercetin; see Section 2.1) compensates for losses of glycosidic precursors.

The α-amylase assay revealed a narrower activity window. MAC-ME achieved an IC_50_ of 1.90 mg/mL, statistically equivalent to UAE-ME (1.94 mg/mL) and marginally more potent than SOE-ME (2.03 mg/mL). All extracts were markedly less effective than acarbose (0.77 mg/mL).

Overall, the results indicate that enzyme inhibition profiles differ among extracts depending on the extraction method.

### 2.4. Correlations Among Phenolic Compounds and Assays

Pearson analysis disclosed several clear association patterns between extract composition and bio-responses (Table 4). The six antioxidant read-outs formed a tightly knit cluster: every pair of assays correlated positively and strongly (*r* = 0.64–0.97), with the highest linkage found between ABTS and DPPH scavenging (*r* = 0.968) and between FRAP and CUPRAC reducing power (*r* = 0.964). Total phenolic content tracked the same direction, registering very high correlations with DPPH (*r* = 0.993) and ABTS (*r* = 0.985) and substantial correlations with the metal-reducing/chelating tests (*r* = 0.80–0.88), confirming that phenolic load is the principal driver of redox potency in the extracts.

Among individual metabolites, hesperidin, hyperoside, and chlorogenic acid emerged as the most influential markers. Hesperidin displayed near-perfect concordance with FRAP (*r* = 0.993), CUPRAC (*r* = 0.958), and ferrous-ion chelation (*r* = 0.979), while hyperoside and chlorogenic acid followed closely (*r* ≥ 0.94 with at least four antioxidant metrics). Conversely, these same compounds correlated negatively and strongly with AChE inhibition (*r* = −0.896 to −0.958), indicating that extracts richer in these flavonoids achieved lower (i.e., more potent) IC_50_ values against the enzyme.

α-Amylase inhibition associated positively with the ferrous-ion chelation test (*r* = 0.930) and, to a lesser extent, with FRAP and CUPRAC (*r* ≈ 0.79–0.81), hinting that metal-binding phenolics also modulate carbohydrate-digestive enzymes. Tyrosinase inhibition, by contrast, showed weak or inconsistent links with both chemistry and other activities (*r* ≤ 0.58), suggesting that additional, non-phenolic constituents—or aglycone ratios generated during extraction—govern this endpoint.

Overall, the correlation matrix highlights strong associations between major phenolics and antioxidant as well as enzyme inhibition parameters.

### 2.5. In Silico Analyses

In this study, the inhibitory potential of the predominant phytochemicals—chlorogenic acid, hesperidin, and hyperoside—identified in three *P. acarna* extracts was predicted against human pancreatic α-amylase (AAMY), AChE, and tyrosinase-related protein 1 (TYRP1) through molecular docking. Docking scores (kcal/mol) calculated for re-docked co-crystallised inhibitors were also employed as reference values for comparison with those obtained for the three phytochemicals. Additionally, the co-crystallised inhibitors were re-docked onto AAMY, AChE, and TYRP1, and the most favourable docked pose obtained for each inhibitor was superposed with its corresponding co-crystallised conformation. The docking protocol was thereby validated by confirming that the associated root mean squared deviation (RMSD) values were 2 Å or lower (Table 5).

**Figure 5:**
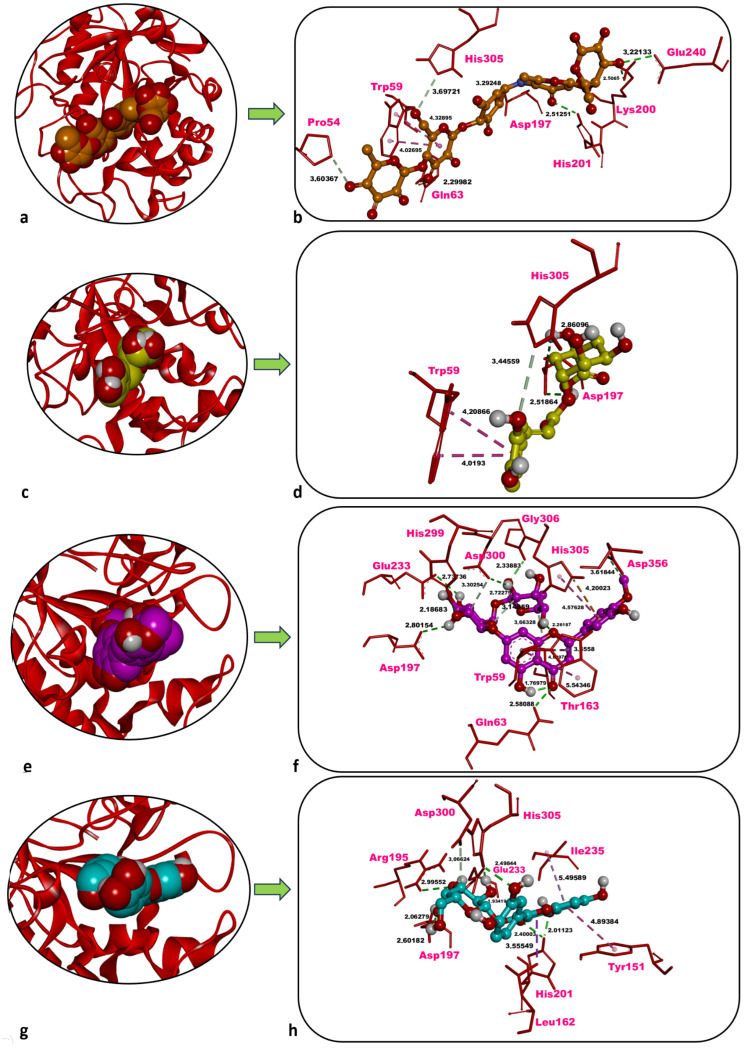
Overview and zoom-in depictions of the top-ranked docking poses for acarbose (inhibitor) (**a**,**b**), chlorogenic acid (**c**,**d**), hesperidin (**e**,**f**), and hyperoside (**g**,**h**) within the catalytic pocket of AAMY. Left panels: three-dimensional representations showing AAMY as a ribbon and each ligand in CPK format. Right panels: magnified interaction diagrams illustrating ligand contacts inside the active site. Conventional hydrogen bonds are denoted by green dashed lines; carbon–hydrogen interactions by light green dashed lines; hydrophobic contacts by light and dark purple dashed lines; and electrostatic interactions by orange dashed lines. Distances for non-bonded contacts (Å) are displayed in bold black. Images generated with Discovery Studio v16. CPK = Corey–Pauling–Koltun.

Acarbose, the co-crystallised inhibitor of AAMY, yielded a docking score of −10.03 kcal/mol. Five classical hydrogen (H) bonds were formed with Gln63 (2.30 Å), Asp197 (3.29 Å), Lys200 (2.51 Å), His201 (2.51 Å), and Glu240 (3.22 Å). Two non-classical carbon–hydrogen bonds were identified with Pro54 (3.60 Å) and His305 (3.70 Å). Hydrophobic contacts occurred with Trp59 (4.03 Å, 4.33 Å) (Table 5, Figure 5a,b).

Chlorogenic acid docked to human pancreatic AAMY displayed a docking score of −7.62 kcal/mol, which is less favourable than that of the co-crystallised inhibitor acarbose (−10.03 kcal/mol). Two classical H-bonds were formed with Asp197 (2.52 Å, 2.86 Å), as well as one non-classical carbon–hydrogen bond which was observed with His305 (3.45 Å). Furthermore, hydrophobic contacts occurred with Trp59 (4.02 Å, 4.21 Å) (Table 5, Figure 5c,d).

Hesperidin showed a highly favourable docking score of −9.58 kcal/mol against human pancreatic AAMY, only slightly less favourable than that of the co-crystallised inhibitor acarbose (−10.03 kcal/mol). Six classical H-bonds were formed with Gln63 (2.58 Å), Asp197 (2.80 Å), Glu233 (2.74 Å), His299 (2.19 Å), Asp300 (2.72 Å), and Gly306 (2.34 Å). Three non-classical carbon–hydrogen bonds were observed with Thr163 (3.66 Å), Asp300 (3.30 Å), and Asp356 (3.62 Å). Moreover, hydrophobic contacts occurred with Trp59 (3.56 Å, 4.89 Å, 5.54 Å) and His305 (4.58 Å), and one electrostatic interaction was detected with His305 (4.20 Å) (Table 5, Figure 5e,f).

Hyperoside showed a docking score of −8.23 kcal/mol against human pancreatic AAMY, less favourable than that of the co-crystallised inhibitor acarbose (−10.03 kcal/mol). Five classical H-bonds were formed with Arg195 (3.00 Å), Asp197 (2.06 Å), His201 (2.01 Å, 2.40 Å), Glu233 (1.93 Å), and His305 (2.50 Å). One non-classical carbon–hydrogen bond was observed with Asp300 (3.07 Å). Hydrophobic contacts occurred with Tyr151 (4.89 Å), Leu162 (3.56 Å), and Ile235 (5.50 Å) (Table 5, Figure 5g,h).

**Figure 6:**
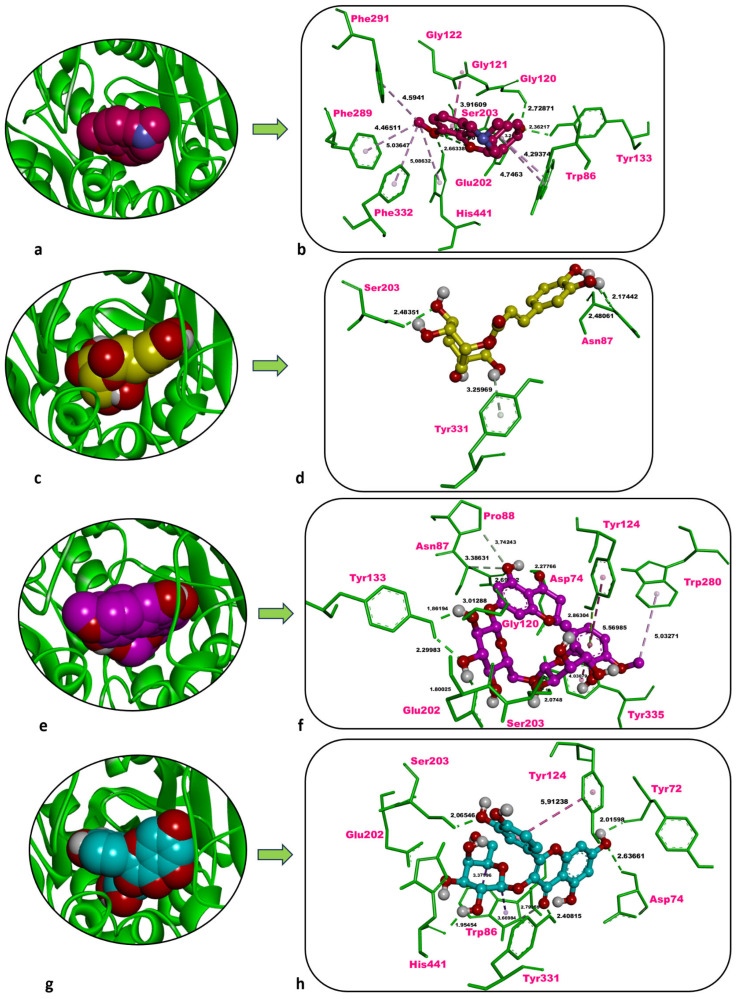
Overview and zoom-in depictions of the top-ranked docking poses for galantamine (inhibitor) (**a**,**b**), chlorogenic acid (**c**,**d**), hesperidin (**e**,**f**), and hyperoside (**g**,**h**) within the catalytic pocket of AChE. Left panels: three-dimensional representations showing AChE as a ribbon and each ligand in CPK format. Right panels: magnified interaction diagrams illustrating ligand contacts inside the active site. Conventional hydrogen bonds are denoted by green dashed lines; carbon–hydrogen interactions by light green dashed lines; pi-lone pair interactions by bright yellow-green; and hydrophobic contacts by light and dark purple dashed lines. Distances for non-bonded contacts (Å) are displayed in bold black. Images generated with Discovery Studio v16. CPK = Corey–Pauling–Koltun.

Galantamine, the co-crystallised inhibitor of human AChE, showed a docking score of −10.46 kcal/mol against the enzyme, along with six classical H-bonds with Gly120 (2.73 Å), Tyr133 (2.36 Å), Glu202 (3.22 Å), Ser203 (2.05 Å, 2.90 Å), and His441 (2.66 Å). Hydrophobic contacts of galantamine, on the other hand, were formed with Trp86 (4.29 Å, 4.75 Å), Gly121 (3.92 Å), Gly122 (3.92 Å), Phe289 (4.47 Å), Phe291 (4.59 Å), Phe332 (5.04 Å), and His441 (5.09 Å) (Table 5, Figure 6a,b).

Chlorogenic acid interacted with human AChE with a docking score of −7.85 kcal/mol, less favourable than that of the inhibitor galantamine (−10.46 kcal/mol). Three classical H-bonds were formed with Asn87 (2.17 Å, 2.48 Å) and Ser203 (2.48 Å), and one non-classical carbon–hydrogen bond was identified with Tyr331 (3.26 Å) (Table 5, Figure 6c,d).

Hesperidin showed a docking score of −5.91 kcal/mol against human AChE, significantly weaker than that of the inhibitor galantamine (−10.46 kcal/mol). Seven classical H-bonds were formed with Asp74 (2.28 Å), Asn87 (2.69 Å), Gly120 (3.01 Å), Tyr133 (1.86 Å, 2.30 Å), Glu202 (1.80 Å), and Ser203 (2.07 Å). Two non-classical carbon–hydrogen bonds were observed with Asn87 (3.39 Å) and Pro88 (3.74 Å). Moreover, hydrophobic contacts occurred with Tyr124 (5.57 Å), Trp280 (5.03 Å), and Tyr335 (4.03 Å), and a π-lone-pair interaction was detected with Tyr124 (2.86 Å) (Table 5, Figure 6e,f).

Hyperoside bound to human AChE with a docking score of −8.43 kcal/mol, weaker than galantamine (−10.46 kcal/mol) yet still indicative of strong ligand binding. Five classical H-bonds were formed with Tyr72 (2.02 Å), Asp74 (2.64 Å), Ser203 (2.07 Å), Tyr331 (2.41 Å), and His441 (1.95 Å), one non-classical carbon–hydrogen bond was observed with Trp86 (2.79 Å). Furthermore, hydrophobic contacts of hyperoside were observed with Trp86 (3.38 Å, 3.67 Å) and Tyr124 (5.91 Å) (Table 5, Figure 6g,h).

**Figure 7:**
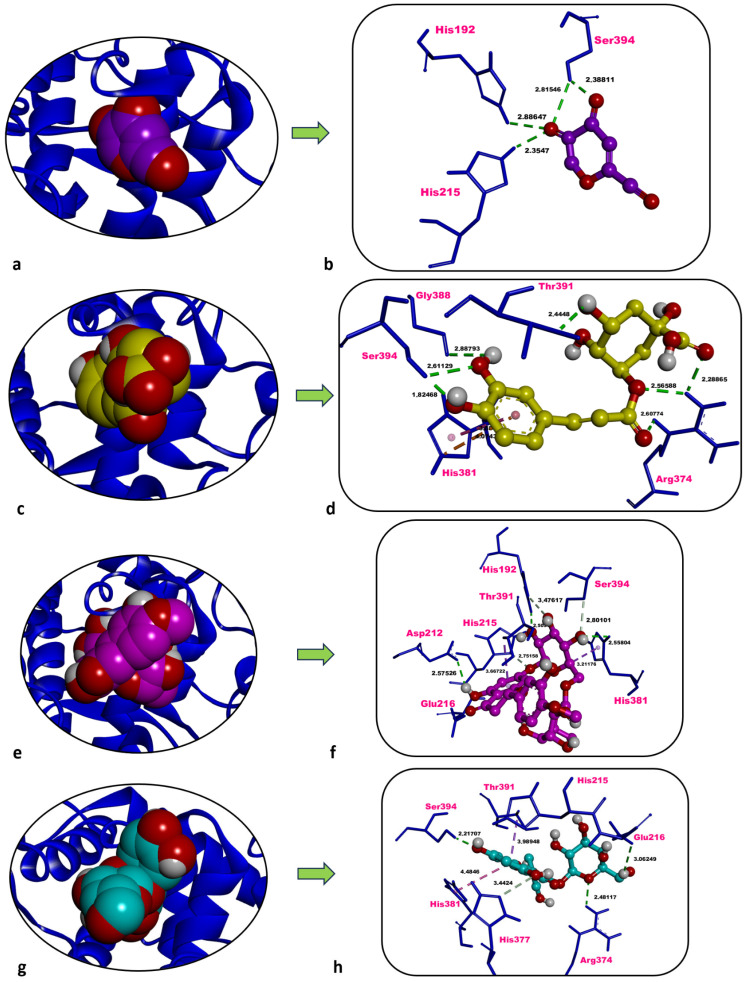
Overview and zoom-in depictions of the top-ranked docking poses for kojic acid (inhibitor) (**a**,**b**), chlorogenic acid (**c**,**d**), hesperidin (**e**,**f**), and hyperoside (**g**,**h**) within the catalytic pocket of TYRP1. Left panels: three-dimensional representations showing TYRP1 as a ribbon and each ligand in CPK format. Right panels: magnified interaction diagrams illustrating ligand contacts inside the active site. Conventional hydrogen bonds are denoted by green dashed lines; carbon–hydrogen interactions by light green dashed lines; electrostatic interactions by orange dashed lines; and hydrophobic contacts by light and dark purple dashed lines. Distances for non-bonded contacts (Å) are displayed in bold black. Images generated with Discovery Studio v16. CPK = Corey–Pauling–Koltun.

Kojic acid, the co-crystallised inhibitor of human TYRP1, showed a docking score of −6.00 kcal/mol against TYRP1. Kojic acid formed four classical H-bonds with His192 (2.89 Å), His215 (2.35 Å), and Ser394 (2.82 Å, 2.39 Å), with no other non-bonded interaction type (Table 5, Figure 7a,b).

Chlorogenic acid interacted with human TYRP1 with a docking score of −6.75 kcal/mol, more favourable than that of the inhibitor kojic acid (−6.00 kcal/mol). Seven classical H-bonds were formed with Arg374 (2.29 Å, 2.57 Å, 2.61 Å), Gly388 (2.89 Å), Thr391 (2.44 Å), and Ser394 (1.82 Å, 2.61 Å). A hydrophobic contact with His381 (3.88 Å) and an electrostatic interaction with the same residue (4.07 Å) were also identified (Table 5, Figure 7c,d).

Docking of hesperidin against human TYRP1 resulted in an energetically unfavourable docking score of +1.80 kcal/mol, markedly less favourable than that of the inhibitor kojic acid (−6.00 kcal/mol). This positive docking score indicates that hesperidin does not form a stable complex with TYRP1 and therefore cannot be considered a potential inhibitor of this target under the applied in silico conditions (Table 5, Figure 7e,f).

Hyperoside interacted with human TYRP1 with a docking score of −7.55 kcal/mol, prominently more favourable and promising than that of the inhibitor kojic acid (−6.00 kcal/mol). Three classical H-bonds were formed with Glu216 (3.06 Å), Arg374 (2.48 Å), and Ser394 (2.22 Å), one non-classical carbon–hydrogen bond was observed with His377 (3.44 Å), along with some hydrophobic contacts which formed with His381 (4.48 Å) and Thr391 (3.99 Å) (Table 5, Figure 7g,h).

Chlorogenic acid and hyperoside exhibited favourable binding affinities toward TYRP1, whereas hesperidin showed an unfavourable (positive) docking score, indicating a lack of inhibitory potential toward this enzyme. Hesperidin produced the most promising score for AAMY, whereas hyperoside showed the top scores for both AChE and TYRP1, matching or surpassing the binding energies of the respective reference inhibitors. Consequently, the literature comparison is centred on these two major phytochemicals of *P. acarna*.

## 3. Discussion

### 3.1. Phenolic Composition and Extraction Selectivity

The present study furnishes the first quantitative, extraction-dependent LC–ESI–MS/MS fingerprint of *P. acarna* aerial parts and, in doing so, extends a phytochemical record that has so far relied on qualitative spot reports. Earlier work isolated a handful of flavones—gossypetin-8,3′,4′-trimethyl ether, luteolin methyl ethers, and related congeners—from Greek collections of the species [11], while a follow-up investigation identified ten glycosylated phenolics (e.g., hyperoside, linarin, pectolinarin) without providing concentration data [12]. By quantifying twenty-four low- and medium-polarity metabolites, we close this knowledge gap and show that *P. acarna* is far richer and more chemotypically plastic than the early spot screens implied.

Total phenolic values obtained from the present study (especially for MAC-ME), after calculation by applying the conversion per 100 g plant on a dry weight basis, are in line with the range of 428–752 mg GAE 100 g/DW reported for *P. acarna* cypselae and *Cardueae taxa* from the same family [4], confirming that the vegetative tissues studied here are an equally competitive antioxidant reservoir. The unexpectedly uniform total flavonoid titres across extraction regimes suggest that the bulk flavonoid pool is dominated by thermally robust constituents—a view borne out by the persistence of rutin-equivalent signals even under Soxhlet reflux. In addition, although ultrasound-assisted extraction is widely reported to enhance mass transfer and extraction efficiency, its effectiveness is highly matrix- and compound-dependent. In the present case, the lack of a significant increase in flavonoid content in UAE-ME indicates that these compounds were already efficiently extracted by conventional maceration, and that cavitation effects did not substantially improve their release. Furthermore, ultrasound-induced localised energy input may promote partial degradation or structural modification of certain flavonoids, counterbalancing potential gains in extraction efficiency.

At the individual-metabolite level, three findings are particularly noteworthy. First, hesperidin emerged as the major component of all three extracts. To our knowledge, no previous *P. acarna* study has detected, let alone quantified, this citrus-type flavanone. Because hesperidin is scarcely mentioned in Cardueae chemistry tables, its high abundance here expands the tribe’s recognised metabolic repertoire and may underlie some of the neuroprotective and vasculotropic claims associated with the plant in folk medicine [13].

Second, maceration clearly outperformed UAE and Soxhlet in conserving medium-polarity caffeoylquinic acids and flavonol glycosides. Room-temperature maceration retained 5135 µg/g chlorogenic acid and 3025 µg/g hyperoside, whereas UAE and SOE suffered 20–45% attrition. The data agree with kinetic models showing that prolonged thermal or cavitational stress accelerates hydrolysis of ester and glycosidic bonds, favouring aglycone build-up at the expense of conjugates [14].

Third, Soxhlet extraction almost doubled the quercetin concentration relative to the macerate (10.6 vs. 5.9 µg/g), supporting the hypothesis that reflux-mediated deglycosylation liberates free flavonols from their 3-*O*-glycosides [15]. Such in-process hydrolysis, while boosting aglycones that are more potent enzyme inhibitors, concurrently erodes the glycoside pool—an observation that helps explain the divergent bioactivity profiles dissected later.

From a chemotaxonomic standpoint, the dominance of hesperidin and chlorogenic acid places *P. acarna* at the phenolic intersection of two lineages: the flavanone-rich *Citrus* clade, in which hesperidin is routinely the prevailing flavonoid [16], and the caffeoyl-quinic-acid-rich thistles of tribe Cardueae, such as globe artichoke, where chlorogenic acid is the principal phenolic constituent [17]. This hybrid profile may trace back to the species’ xerothermic habitat, where dual protection against UV stress (hesperidin) and oxidative burst (chlorogenic acid) would be advantageous. The consistent presence of hyperoside, pectolinarin, and linarin corroborates the 1995 report [12], but our dataset provides the first numerical hierarchy among these markers, thereby supplying a quantitative baseline for future authentication or valorisation efforts.

Finally, by benchmarking three extraction paradigms, we demonstrate that process variables modulate the quality of *P. acarna* extracts as much as they do crude yield. Maceration maximizes conjugate recovery; Soxhlet sacrifices heat-labile glycosides yet enriches free aglycones; UAE offers a pragmatic compromise, retrieving ~80% of the maceration phenolic spectrum in one-twentieth of the processing time.

Importantly, the observed differences in biological performance among extracts obtained with the same solvent can be attributed to variations in extraction energy input, temperature, and mass transfer mechanisms. While the solvent system remained constant, maceration preserves structurally intact, glycosylated phenolics due to mild conditions; Soxhlet extraction promotes thermal hydrolysis and deglycosylation, increasing aglycone content; and ultrasound-assisted extraction enhances cell disruption through cavitation but may simultaneously induce localised degradation. These compositional shifts directly influence biological activity, as glycosides and aglycones differ in polarity, stability, and enzyme-binding affinity, thereby leading to distinct antioxidant and enzyme inhibitory profiles despite identical assay conditions.

These insights position the current work as a methodological template for optimising thistle-derived ingredients in nutraceutical or cosmeceutical pipelines.

In addition to compositional differences, solvent selectivity and extraction yield should be considered together when interpreting bioactivity outcomes. Although all extracts were obtained using methanol, the effective selectivity of the solvent is modulated by extraction conditions such as temperature, duration, and energy input, which influence solvent penetration and compound solubilisation. In the present study, Soxhlet extraction yielded the highest crude extract percentage, whereas maceration produced extracts with lower yield but higher phenolic concentration and superior antioxidant performance. This indicates that extraction yield alone is not a reliable predictor of biological activity. Instead, selective enrichment of bioactive constituents—particularly phenolic glycosides in maceration and aglycones in Soxhlet—appears to be the determining factor. Therefore, optimisation of extraction protocols should prioritise chemical selectivity and target compound recovery rather than maximising total yield, as higher yield may reflect the co-extraction of inactive or less active matrix components.

### 3.2. Antioxidant Capacity in Relation to Extraction Method

To our knowledge, the antioxidant capacity of *P. acarna* has only once been quantified before, and that single report dealt exclusively with the cypselae (fruits). Kurt et al. [4] extracted the seeds with 80% methanol and expressed activity as Trolox equivalents: DPPH 144.28 µmol TE/g dw and FRAP 82.57 µmol TE/g dw. Because our study employed IC_50_/EC_50_ formats on aerial-part extracts, direct numerical comparison is not straightforward; nonetheless, the macerated extract’s DPPH IC_50_ of 1.85 mg/mL and FRAP EC_50_ of 0.91 mg/mL rank in the middle of the activity range reported for well-studied Cardueae such as *Cirsium* spp. and *Centaurea* spp. when the same assay conditions are applied. More importantly, we deliver the first side-by-side evaluation of six complementary redox assays and three extraction technologies for *P. acarna*, revealing that room-temperature maceration safeguards antioxidant potential far better than Soxhlet or ultrasound processing.

The genus *Picnomon* is monotypic; consequently, no interspecific comparison within the genus is possible. However, the single earlier seed study [4] offers an indirect internal control: our macerated aerial extract shows a 23% lower total phenolic content than the cypsela extract (on a gallic-acid basis), yet its radical- and metal-reducing potencies are of the same order of magnitude. This suggests that leaf and stem matrices contain a higher proportion of high-efficacy molecules—an inference supported by our LC–ESI–MS/MS fingerprint.

Three dominant constituents—hesperidin, chlorogenic acid, and hyperoside—are plausible drivers of the observed bioactivity. Hesperidin regulates Nrf2-linked antioxidant defences in mammalian and human models and consistently lowers oxidative biomarkers in meta-analysed clinical data [18]. Chlorogenic acid is a benchmark polyphenol whose in vitro and in vivo ROS-quenching, FRAP, and CUPRAC values rival those of trolox, acting partly through Nrf2 and AMPK signalling [19]. Hyperoside (quercetin-3-*O*-galactoside) reduces intracellular ROS and lipid peroxidation in *Saccharomyces* and mammalian cell systems, outperforming quercetin at equimolar concentration [20]. All three molecules occur at milligram-per-gram levels in the macerate, and their combined concentration (~14 mg/g) represents nearly half of the quantified phenolic pool, providing a mechanistic explanation for the extract’s superior DPPH, ABTS, and metal-chelating scores.

Thermal processing clearly re-shaped the antioxidant landscape. Soxhlet treatment almost doubled free quercetin relative to the macerate, a shift that mirrors the thermal deglycosylation of quercetin glucosides reported by Rohn et al. [15] under roasting conditions (180 °C) [15]. While the liberated aglycone is a powerful electron donor, the concurrent loss of glycosides (e.g., hyperoside) explains why the Soxhlet extract never surpassed the macerate in any assay: the gain in quercetin could not compensate for the broad depletion of conjugated flavonols and caffeoylquinates. Ultrasound extraction, by contrast, preserved a larger fraction of the glycoside pool but still inflicted a 20–30% drop in hesperidin and chlorogenic acid, resulting in intermediate antioxidant indices. These findings further support that differences in antioxidant performance are not driven by solvent polarity, but rather by extraction-induced compositional changes, particularly the balance between thermolabile glycosides and more stable aglycones.

Taken together, our data position *P. acarna* aerial parts as a credible source of multifunctional redox modulators and demonstrate that the extraction strategy is a decisive quality determinant. The study (i) reports the first multi-parametric antioxidant profile for vegetative tissues of the species, (ii) dissects how process variables shift the balance between glycosidic and aglycone phenolics, and (iii) links those shifts to functional read-outs across six assay platforms. These insights provide a rational basis for tailoring *P. acarna* extracts to nutraceutical or cosmeceutical applications where either broad-spectrum antioxidant protection (maceration) or aglycone-enriched enzyme inhibition (mild reflux) is desired.

### 3.3. Enzyme Inhibition Patterns and Phytochemical Basis

The present work is the first to quantify the inhibitory effects of *P. acarna* extracts on AChE, tyrosinase, and α-amylase, expanding a phytochemical record that until now lacked any functional enzymology. Because no earlier bioassays on *P. acarna* could be located, our IC_50_ values define the first numerical benchmark for the species.

Comparable data from other Cardueae thistles underscore the distinctive potency of *P. acarna*. A methanolic leaf macerate of *Carduus crispus* inhibited AChE by 60% at 1 µg/mL (Ellman assay) but yielded no IC_50_, precluding direct comparison; nevertheless, its activity drops sharply above that dose, giving an extrapolated IC_50_ close to 2 mg/mL—slightly weaker than the Soxhlet extract reported here [21]. For tyrosinase, *Cirsium japonicum* var. *maackii* supplies the strongest reference point: a luteolin-5-O-β-d-glucopyranoside isolated from the inflorescences suppressed mushroom tyrosinase with an IC_50_ of 2.95 µg/mL, whereas the corresponding crude extract was far less active (≈2 mg/mL) [22]. Against that backdrop, the present Soxhlet IC_50_ of 1.48 mg/mL ranks at the high end of crude-extract efficacy, demonstrating that *P. acarna* can rival better-known thistles despite being assessed here in its unrefined form.

Process-dependent shifts in potency parallel the compositional changes described in Section 3.1. Soxhlet reflux, which nearly doubled free quercetin, delivered the tightest AChE and tyrosinase IC_50_ values, supporting literature that aglycones outperform their glycosylated parents at these targets. Chlorogenic acid, detected at milligram-per-gram levels in the macerate and retained at moderate levels in Soxhlet, is itself a mixed-type AChE blocker with IC_50_ values around 40 µg/mL in purified systems [23], while quercetin liberated during reflux is an established competitive ligand for tyrosinase copper sites. Conversely, the slightly stronger α-amylase suppression recorded for the macerated extract aligns with its higher content of hesperidin and hyperoside, two flavonoids that inhibit the enzyme in the sub-milligram-per-millilitre range (IC_50_ ≈ 0.43–0.49 mg/mL) by occupying the catalytic groove and perturbing substrate binding [24,25].

The contribution of the dominant metabolites to the observed bioactivity is therefore plausible. Hesperidin shows sub-micromolar affinity for human AChE in silico and produces measurable enzyme knockdown in cell models [26]; chlorogenic acid slows AChE turnover and attenuates α-amylase and tyrosinase in complementary assays [27,28]; and hyperoside couples potent α-amylase inhibition with moderate AChE and tyrosinase effects [26]. The enrichment of aglycones under Soxhlet conditions further explains why that fraction excels against AChE and tyrosinase, whereas the glycoside-rich macerate is marginally superior toward α-amylase.

In sum, the enzyme-inhibitory landscape of *P. acarna* is both process-sensitive and chemically rational: mild maceration conserves glycosylated flavonoids that favour carbohydrate-digestive targets, whereas controlled reflux enhances aglycones that are better suited to neuromodulatory and melanogenic enzymes. These insights, reported here for the first time, position *P. acarna* as a versatile candidate for nutraceutical formulations aimed at neuroprotection, skin depigmentation, or post-prandial glycaemic control, depending on the extraction route employed. Thus, even under identical assay conditions, the differential enzyme inhibition profiles arise primarily from extraction-driven alterations in phytochemical composition, rather than experimental variability.

### 3.4. Molecular Docking Insights into Bioactivity

Hesperidin yielded docking scores of −9.60, −8.22, and −8.60 kcal/mol against human AAMY and more intensely formed multiple H-bonds with the catalytic triad residues Asp197, Glu233, and Asp300 in the enzyme’s active site [29,30]. These observations align with the current study, in which hesperidin not only interacted with the catalytic triad but also established additional non-bonded contacts with Trp59, Gln63, and His305—residues commonly engaged by the co-crystallised inhibitor acarbose. The corresponding docking score of −9.58 kcal/mol further highlights the ligand’s strong affinity against AAMY. Collectively, the data position hesperidin as a promising competitive inhibitor of starch hydrolysis.

Hyperoside isolated from *Bellardia trixago* displayed a docking score of −10.0 kcal/mol against mouse AChE and was stabilised by a network of non-bonded interactions with active-site residues Asp74, Trp86, Gly121, Gly122, Tyr124, Tyr133, Glu202, Phe295, Arg296, Tyr341, and His447 [31]. In the present study, the same phytochemical displayed a binding affinity of −8.43 kcal/mol toward human AChE, forming non-bonded contacts with Tyr72, Asp74, Trp86, Tyr124, Ser203, Tyr331, and His441. Notably, Asp74, Trp86, and Tyr124 represent shared interaction hotspots in both species. Thus, these findings indicate that hyperoside possesses a strong affinity for AChE and merits further optimisation as a lead compound.

Hyperoside isolated from *Astragalus melanophrurius* exhibited a binding energy of −7.78 kcal/mol toward TYRP1 and engaged predominantly H-bond-mediated interactions with His192, Asp212, His215, Arg321, Arg374, His381, Thr391, and Ser394 [32]. In the present study, hyperoside bound to human TYRP1 with an energy of −7.55 kcal/mol and was stabilised through non-bonded contacts with Glu216, Arg374, His377, His381, Thr391, and Ser394. Notably, its more favourable affinity relative to the reference inhibitor kojic acid (−7.55 kcal/mol vs. −6.00 kcal/mol) may provide molecular evidence that hyperoside could also serve as a potent tyrosinase inhibitor candidate.

In contrast to its strong binding affinity toward AAMY, hesperidin did not exhibit a favourable interaction with TYRP1 in the present study, as reflected by its positive docking score (+1.80 kcal/mol). This result indicates that hesperidin is unlikely to contribute to the observed tyrosinase inhibitory activity, which is more plausibly associated with other constituents such as hyperoside and chlorogenic acid.

Nevertheless, molecular docking has intrinsic methodological limitations, as it mainly describes the initial and relatively static recognition events between small molecules and their macromolecular targets, without fully accounting for protein flexibility or the conformational ensembles sampled during biochemical processes. Accordingly, docking predictions should be complemented by molecular dynamics (MD) simulations, which enable a more detailed assessment of the time-dependent structural behaviour, interaction persistence, and thermodynamic stability of ligand–protein complexes.

## 4. Materials and Methods

### 4.1. Plant Material

Aerial portions of *P. acarna* were harvested on 17 May 2024 from clearings within *Pinus brutia* stands, field margins, and roadside verges near Gülağzı village, Menteşe–Muğla, Türkiye (640 m a.s.l.; 37°08′27″ N, 28°21′26″ E). Specimens were authenticated by Dr Olcay Ceylan (Department of Biology, Muğla Sıtkı Koçman University), and voucher material has been deposited in the university herbarium under the accession O.2152.

### 4.2. Preparation of Methanolic Extracts

Three extraction protocols were applied following Zengin et al. [33]. For maceration, 5 g of air-dried plant powder were immersed in 100 mL of methanol for 24 h (1:20 *w*/*v*). Ultrasound-assisted extraction employed the same solvent and ratio but utilised a sonication bath for 60 min. Soxhlet extraction was performed for 6 h with methanol under standard Soxhlet conditions. All filtrates were concentrated under reduced pressure at 40 °C to dryness, and the dried residues were kept at 4 °C in the dark until use. Extraction efficiencies for MAC, SOE, and UAE were 10.78%, 15.00%, and 10.19%, respectively.

### 4.3. Phenolic Profiling

Total phenolic and flavonoid contents were quantified spectrophotometrically as described by Zengin et al. [34]. Individual phytochemicals were identified and quantified by a previously validated LC–ESI–MS/MS method [35]; analytical performance parameters are presented in Supplementary Tables S1 and S2.

### 4.4. Assessment of Biological Activities

Antioxidant capacity was evaluated using the phosphomolybdenum, 2,2-diphenyl-1-picrylhydrazyl (DPPH), 2,2′-azinobis-(3-ethylbenzothiazoline-6-sulfonic acid) (ABTS), cupric reducing antioxidant capacity (CUPRAC), ferric reducing antioxidant power (FRAP), and ferrous-ion chelation assays [34,36,37,38,39]. Enzyme inhibition against tyrosinase, α-amylase, and acetylcholinesterase followed the protocol of Ozer et al. [40]. Full methodological details are provided in the Supplementary File.

### 4.5. Molecular Docking Analysis

To corroborate the enzyme-inhibitory profiles of the methanolic extracts, we undertook a structure-based molecular docking assessment using three abundant constituents of *P. acarna*—chlorogenic acid, hesperidin, and hyperoside—against human pancreatic α-amylase, acetylcholinesterase, and tyrosinase-related protein-1. Ligand geometries were constructed in ChemOffice 19.1 and energy-minimized in Avogadro 1.2.0, whereas the high-resolution receptor structures (PDB IDs: 1B2Y, 4EY6, and 5M8M) were prepared through standard protein-processing procedures, including the removal of crystallographic artifacts, loop rebuilding, and pH-dependent protonation [27,41,42]. Partial charges and docking input files were assigned and generated using AutoDockTools 1.5.7, and semi-flexible docking calculations were carried out with AutoDock Vina 1.2.7 at an exhaustiveness level of 64, with the search grids centered on the coordinates of the co-crystallized reference inhibitors [28,43]. All three ligands exhibited favorable binding free energies, ranging from −7.3 to −10.1 kcal/mol, and reproduced key hydrogen-bonding and π-stacking interactions observed for the reference inhibitors, supporting the view that these metabolites may contribute to the extract-mediated inhibition of carbohydrate-metabolizing, cholinergic, and melanogenic enzymes [44,45].

### 4.6. Statistical Treatment

The relative antioxidant capacity index (RACI) was calculated according to Sun and Tanumihardjo [46]. All experiments were performed in triplicate (*n* = 3), and results are expressed as mean ± standard deviation (SD). Additional statistical procedures are outlined in the Supplementary File.

## 5. Conclusions

Room-temperature maceration preserved glycosylated phenolics in *P. acarna*, yielding the richest hesperidin–hyperoside–chlorogenic acid triad, the strongest multi-assay antioxidant performance, and the most favourable global RACI score. Soxhlet extraction, while depleting these glycosides, liberated aglycone flavonols and consequently produced extracts that excelled at AChE and tyrosinase inhibition, whereas ultrasound-assisted extraction offered an intermediate profile. Correlation and docking analyses converged on the same mechanistic drivers: phenolic load—particularly the three dominant metabolites—accounted for >80% of the variance in redox indices and cholinesterase blockade, and in silico modelling identified hesperidin as a top-scoring ligand for α-amylase, while hyperoside (but not hesperidin) demonstrated the most favourable binding toward TYRP1. Collectively, these findings establish *P. acarna* as a versatile phytochemical source whose bioactivity spectrum can be modulated by simple changes in extraction energy and solvent contact time.

Going forward, three avenues merit priority. First, well-designed cell-based assays and in vivo studies are required to confirm whether the in vitro and in silico activities observed in this study can be translated into biologically relevant effects under physiological conditions. In particular, cellular models focusing on oxidative stress, neuroprotection, and melanogenesis, as well as animal models evaluating enzyme inhibition and systemic antioxidant responses, would provide critical insight into the bioefficacy, bioavailability, and safety of *P. acarna* extracts. Second, fractionation of aglycone- and glycoside-enriched sub-extracts, followed by synergy testing, could clarify cooperative effects hinted at by the correlation matrix. Finally, integrating green extraction technologies (e.g., deep-eutectic solvents or pressurised water) with formulation approaches such as nanoencapsulation may enhance bioavailability while lowering solvent and energy footprints. Addressing these questions will accelerate the progression of *P. acarna* from a regional edible weed to a credible nutraceutical or adjunct phytotherapeutic candidate. Overall, this study provides the first comprehensive phytochemical and bioactivity evaluation of *P. acarna* aerial extracts, offering new insights into its potential as a natural source of bioactive molecules.

## Figures and Tables

**Table 1:** Concentration (µg/g extract) of selected phenolic compounds in the methanol extracts from *P. acarna*.

Compounds	MAC-ME	SOE-ME	UAE-ME
Hesperidin	5896 ± 13 ^a^	4372 ± 65 ^c^	4722 ± 80 ^b^
Chlorogenic acid	5135 ± 12 ^a^	4139 ± 39 ^b^	3800 ± 13 ^c^
Hyperoside	3025 ± 12 ^a^	2308 ± 50 ^b^	2321 ± 15 ^b^
Ferulic acid	902 ± 2 ^a^	710 ± 1 ^c^	821 ± 11 ^b^
4-Hydroxybenzoic acid	484 ± 1 ^a^	416 ± 6 ^c^	459 ± 4 ^b^
Pinoresinol	247 ± 3 ^a^	210 ± 4 ^b^	234 ± 4 ^a^
Luteolin	236 ± 3 ^a^	185 ± 1 ^b^	170 ± 2 ^c^
Vanillin	136 ± 2 ^a^	94.1 ± 0.1 ^c^	107 ± 1 ^b^
Apigenin	108 ± 1 ^a^	78.2 ± 0.5 ^b^	79.6 ± 0.1 ^b^
p-Coumaric acid	66.5 ± 1.1 ^a^	63.8 ± 0.5 ^ab^	59.7 ± 1.9 ^b^
Protocatechuic acid	62.6 ± 1.0 ^a^	57.1 ± 0.2 ^b^	52.8 ± 0.5 ^c^
Apigenin 7-glucoside	59.1 ± 0.2 ^a^	42.1 ± 0.2 ^b^	37.4 ± 0.1 ^c^
Caffeic acid	48.9 ± 0.1 ^a^	37.5 ± 0.3 ^b^	36.5 ± 0.1 ^c^
Luteolin 7-glucoside	60.2 ± 0.7 ^a^	32.8 ± 0.4 ^c^	38.6 ± 0.3 ^b^
Taxifolin	30.3 ± 0.6 ^a^	23.2 ± 0.2 ^b^	23.7 ± 0.6 ^b^
2,5-Dihydroxybenzoic acid	15.9 ± 0.2 ^a^	17.7 ± 0.5 ^a^	15.6 ± 0.6 ^a^
Syringic acid	21.9 ± 1.3 ^a^	16.7 ± 0.6 ^b^	17.4 ± 0.2 ^b^
Eriodictyol	19.9 ± 0.4 ^a^	15.8 ± 0.2 ^c^	16.9 ± 0.1 ^b^
Gallic acid	11.8 ± 0.3 ^a^	11.5 ± 0.4 ^a^	7.86 ± 0.49 ^b^
Quercetin	5.93 ± 0.06 ^c^	10.56 ± 0.15 ^a^	6.75 ± 0.03 ^b^
Kaempferol	8.46 ± 0.40 ^a^	7.87 ± 0.19 ^a^	6.48 ± 0.21 ^b^
2-Hydroxycinnamic acid	1.37 ± 0.10 ^c^	3.19 ± 0.01 ^a^	2.44 ± 0.07 ^b^
Sinapic acid	1.38 ± 0.07 ^a^	0.91 ± 0.01 ^b^	0.87 ± 0.02 ^b^
(−)-Epicatechin	0.75 ± 0.02 ^a^	0.52 ± 0.03 ^b^	0.82 ± 0.06 ^a^
Pyrocatechol	nd	nd	nd
3,4-Dihydroxyphenylacetic acid	nd	nd	nd
(+)-Catechin	nd	nd	nd
3-Hydroxybenzoic acid	nd	nd	nd
Verbascoside	nd	nd	nd
Rosmarinic acid	nd	nd	nd

Values indicated by the same superscripts (a–c) are not significantly different according to Tukey’s post hoc test at the 5% significance level. nd: Not detected.

**Table 2:** Antioxidant activities of the methanol extracts from *P. acarna*.

Assays	MAC-ME	SOE-ME	UAE-ME	EDTA	Trolox
Ferrous ion chelating (IC_50_: mg/mL)	1.20 ± 0.01 ^b^	1.77 ± 0.02 ^c^	1.53 ± 0.12 ^c^	0.036 ± 0.004 ^a^	-
DPPH radical scavenging (IC_50_: mg/mL)	1.85 ± 0.02 ^b^	2.16 ± 0.03 ^c^	2.60 ± 0.04 ^d^	-	0.057 ± 0.002 ^a^
ABTS radical cation scavenging (IC_50_: mg/mL)	1.45 ± 0.004 ^b^	1.73 ± 0.02 ^c^	1.86 ± 0.01 ^d^	-	0.11 ± 0.003 ^a^
CUPRAC reducing power (EC_50_: mg/mL)	1.20 ± 0.01 ^b^	1.34 ± 0.03 ^c^	1.34 ± 0.02 ^c^	-	0.12 ± 0.01 ^a^
FRAP reducing power (EC_50_: mg/mL)	0.91 ± 0.01 ^b^	1.06 ± 0.004 ^d^	1.03 ± 0.005 ^c^	-	0.045 ± 0.004 ^a^
Phosphomolybdenum (EC_50_: mg/mL)	1.74 ± 0.13 ^b^	2.09 ± 0.06 ^c^	1.99 ± 0.02 ^bc^	-	0.49 ± 0.03 ^a^

TEs and EDTAEs mean trolox and ethylenediaminetetraacetic acid (disodium salt) equivalents, respectively. Values indicated by the same superscripts (a–d) are not significantly different according to Tukey’s post hoc test at the 5% significance level.

**Table 3:** Enzyme inhibition activity of the methanol extracts from *P. acarna*.

Samples	AChE Inhibition (IC_50_: mg/mL)	Tyrosinase Inhibition (IC_50_: mg/mL)	α-Amylase Inhibition (IC_50_: mg/mL)
MAC-ME	1.52 ± 0.10 ^c^	1.54 ± 0.01 ^c^	1.90 ± 0.01 ^b^
SOE-ME	1.21 ± 0.01 ^b^	1.48 ± 0.004 ^b^	2.03 ± 0.02 ^c^
UAE-ME	1.25 ± 0.04 ^b^	1.64 ± 0.03 ^d^	1.94 ± 0.05 ^bc^
Galanthamine	0.0029 ± 0.0002 ^a^	-	-
Kojic acid	-	0.083 ± 0.002 ^a^	-
Acarbose	-	-	0.77 ± 0.02 ^a^

Values indicated by the same superscripts (a–d) are not significantly different according to Tukey’s post hoc test at the 5% significance level.

**Table 4:** Correlation coefficients between phenolic compounds and antioxidant/enzyme inhibition assays.

	TAP	DPPH	ABTS	CUPRAC	FRAP	FICA	AChEIA	TIA	AAIA
DPPH	0.682								
ABTS	0.801	0.968							
CUPRAC	0.844	0.847	0.947						
FRAP	0.883	0.750	0.891	0.964					
FICA	0.885	0.640	0.804	0.938	0.964				
AChEIA	−0.784	−0.764	−0.889	−0.971	−0.966	−0.954			
TIA	−0.080	0.578	0.357	0.092	−0.104	−0.216	0.023		
AAIA	0.798	0.376	0.570	0.789	0.813	0.930	−0.803	−0.424	
Total flavonoid	−0.597	−0.907	−0.836	−0.629	−0.577	−0.398	0.508	−0.625	−0.098
Total phenolic	0.742	0.993	0.985	0.880	0.807	0.694	−0.799	0.493	0.433
Hesperidin	0.921	0.738	0.880	0.958	0.993	0.979	−0.958	−0.113	0.847
Chlorogenic acid	0.811	0.960	0.997	0.941	0.900	0.812	−0.896	0.334	0.567
Hyperoside	0.893	0.856	0.954	0.971	0.978	0.927	−0.955	0.081	0.734
Ferulic acid	0.844	0.424	0.634	0.817	0.908	0.956	−0.856	−0.479	0.940
4-Hydroxybenzoic acid	0.837	0.359	0.575	0.760	0.871	0.930	−0.810	−0.533	0.927

Data show the Pearson Correlation Coefficients between the parameters. TAP: total antioxidant activity by phosphomolybdenum method. AAIA, AChEIA, and TIA: α-amylase, acetylcholinesterase, and tyrosinase inhibition activities, respectively. ABTS and DPPH: ABTS and DPPH radical scavenging activities, respectively. CUPRAC and FRAP: CUPRAC and FRAP reducing power potential, respectively. FICA: Ferrous ion chelating activity.

**Table 5:** Molecular docking results for major *P. acarna* major phytochemicals and reference inhibitors against AAMY, AChE, and TYRP1, showing docking scores (kcal/mol) and detailed ligand–residue contacts within each enzyme active site.

Compound	Molecular Weight (g/mol)	Target Protein (ΔG: kcal/mol)	Docking Score (ΔG: kcal/mol)	RMSD (Root Mean Squared Deviation)	Classical H-Bond	Non-Classical H-Bond (Carbon–Hydrogen, pi-Donor)	Hydrophobic Contact	Electrostatic (pi-Cation, pi-Anion)	Other (pi-Lone Pair)
							π-Sigma, Alkyl, π-Alkyl and π-π Stacked, and π-π T-Shaped İnteraction		
Acarbose (inhibitor)	756.492	AAMY	−10.03	0.89	Gln63 (2.30 Å), Asp197 (3.29 Å), Lys200 (2.51 Å), His201 (2.51 Å), Glu240 (3.22 Å)	Pro54 (3.60 Å), His305 (3.70 Å)	Trp59 (4.03 Å, 4.33 Å)	-	-
Chlorogenic acid	354.32	AAMY	−7.62		Asp197 (2.52 Å, 2.86 Å)	His305 (3.45 Å)	Trp59 (4.02 Å, 4.21 Å)	-	-
Hesperidin	610.58	AAMY	−9.58		Gln63 (2.58 Å), Asp197 (2.80 Å), Glu233 (2.74 Å), His299 (2.19 Å), Asp300 (2.72 Å), Gly306 (2.34 Å)	Thr163 (3.66 Å), Asp300 (3.30 Å), Asp356 (3.62 Å)	Trp59 (3.56 Å, 4.89 Å, 5.54 Å), His305 (4.58 Å)	His305 (4.20 Å)	-
Hyperoside	464.39	AAMY	−8.23		Arg195 (3.00 Å), Asp197 (2.06 Å), His201 (2.01 Å, 2.40 Å), Glu233 (1.93 Å), His305 (2.50 Å)	Asp300 (3.07 Å)	Tyr151 (4.89 Å), Leu162 (3.56 Å), Ile235 (5.50 Å)	-	-
Galantamine (inhibitor)	285.34	AChE	−10.46	0.56	Gly120 (2.73 Å), Tyr133 (2.36 Å), Glu202 (3.22 Å), Ser203 (2.05 Å, 2.90 Å), His441 (2.66 Å)	-	Trp86 (4.29 Å, 4.75 Å), Gly121 (3.92 Å), Gly122 (3.92 Å), Phe289 (4.47 Å), Phe291 (4.59 Å), Phe332 (5.04 Å), His441 (5.09 Å)	-	-
Chlorogenic acid	354.32	AChE	−7.85		Asn87 (2.17 Å, 2.48 Å), Ser203 (2.48 Å)	Tyr331 (3.26 Å)	-	-	-
Hesperidin	610.58	AChE	−5.91		Asp74 (2.28 Å), Asn87 (2.69 Å), Gly120 (3.01 Å), Tyr133 (1.86 Å, 2.30 Å), Glu202 (1.80 Å), Ser203 (2.07 Å)	Asn87 (3.39 Å), Pro88 (3.74 Å)	Tyr124 (5.57 Å), Trp280 (5.03 Å), Tyr335 (4.03 Å)	-	Tyr124 (2.86 Å)
Hyperoside	464.39	AChE	−8.43		Tyr72 (2.02 Å), Asp74 (2.64 Å), Ser203 (2.07 Å), Tyr331 (2.41 Å), His441 (1.95 Å)	Trp86 (2.79 Å)	Trp86 (3.38 Å, 3.67 Å), Tyr124 (5.91 Å)	-	-
Kojic acid (inhibitor)	139.09	TYRP1	−6.00	2.07	His192 (2.89 Å), His215 (2.35 Å), Ser394 (2.82 Å, 2.39 Å)	-	-	-	-
Chlorogenic acid	354.32	TYRP1	−6.75		Arg374 (2.29 Å, 2.57 Å, 2.61 Å), Gly388 (2.89 Å), Thr391 (2.44 Å), Ser394 (1.82 Å, 2.61 Å)	-	His381 (3.88 Å)	His381 (4.07 Å)	-
Hesperidin	610.58	TYRP1	+1.80		His192 (2.51 Å), Asp212 (2.58 Å), His381 (2.56 Å)	His192 (3.48 Å), His215 (2.75 Å), Ser394 (2.80 Å)	His381 (3.21 Å), Thr391 (3.67 Å)	-	-
Hyperoside	464.39	TYRP1	−7.55		Glu216 (3.06 Å), Arg374 (2.48 Å), Ser394 (2.22 Å)	His377 (3.44 Å)	His381 (4.48 Å), Thr391 (3.99 Å)	-	-

AAMY: Human pancreatic alpha-amylase in complex with acarbose (PDB ID: 1B2Y). AChE: human acetylcholinesterase in complex with (−)-galantamine (PDB ID: 4EY6). TYRP1: Human tyrosinase related protein 1 in complex with kojic acid (PDB ID: 5M8M). ΔG: Docking score (kcal/mol) of the top-ranked binding pose of the ligand. Residues formatted in italics and underlined denote critical active site contacts shared by both the major phytochemicals and reference inhibitors during docking. This shared interaction pattern validates the conformational sampling approach of AutoDock Vina v1.2.7.

## Data Availability

Data will be made available on request.

## Supplementary Materials

The following supporting information can be downloaded at https://www.mdpi.com/article/10.3390/molecules31081240/s1. Section S1: Analytical methods applied for phenolic composition, antioxidant and enzyme inhibitory activities; Table S1: LC–ESI–MS/MS parameters and analytical characteristics for the analysis of target analytes by MRM Negative and Positive Ionization Mode; Table S2: Calibration curves and sensitivity properties of the LC–MS/MS method.
